# Evolving epileptic activity under focal rhythmic Delta activity: insights from simultaneous intracranial and scalp EEG

**DOI:** 10.1016/j.cnp.2026.08.001

**Published:** 2026-08-09

**Authors:** Ayumi Sakata, Nobutaka Mukae, Takafumi Shimogawa, Takahiko Mukaino, Kimiaki Hashiguchi, Kazuhisa Kuwabara, Kou Matsumoto, Takahiro Yamaguchi, Taira Uehara, Toshiki Okadome, Yasunari Sakai, Yuri Sonoda, Satoshi Akamine, Pin Fee Chong, Kenta Kajiwara, Eriko Watanabe, Noriko Isobe, Koji Yoshimoto, Takato Morioka, Yuya Kunisaki, Hiroshi Shigeto

**Affiliations:** a Division of Medical Technology, Department of Health Sciences, Graduate School of Medical Sciences, Kyushu University, Fukuoka, Japan; b Department of Clinical Laboratory, Kyushu University Hospital, Fukuoka, Japan; c Department of Neurosurgery, Graduate School of Medical Sciences, Kyushu University, Fukuoka, Japan; d Department of Neurology, Graduate School of Medical Sciences, Kyushu University, Fukuoka, Japan; e Department of Neurology, Kyushu University Hospital, Fukuoka, Japan; f Hashiguchi Neuro Clinic, Fukuoka, Japan; g Department of Neurology, Fukuoka Sanno Hospital, Fukuoka, Japan; h Department of Pediatrics, Graduate School of Medical Sciences, Kyushu University, Fukuoka, Japan; i Department of Neurosurgery, Hachisuga Hospital, Munakata, Japan

**Keywords:** Focal rhythmic delta activity, Intracranial hematoma, Intracranial recording, Electrographic seizures, Electrographic status epilepticus, High-frequency oscillation

## Abstract

**Objective:**

Focal rhythmic delta activity (RDA) is a subtype of lateralized RDA (LRDA). Although several studies have compared scalp and intracranial electroencephalography (EEG) recordings to determine whether LRDA observed on scalp EEG reflects epileptic abnormalities occurring intracranially during the acute phase, to our knowledge, no studies have examined focal RDA. Therefore, we investigated the extent to which focal RDA on scalp EEG during the acute phase reflects epileptic activity observed on intracranial EEG.

**Methods:**

We retrospectively reviewed cases with simultaneous intracranial and scalp EEG (January 2003–August 2024). Patients who developed an intracranial hematoma during electrode placement were identified and included. For each case, we evaluated whether RDA, spikes, polyspikes, electrographic seizures (ESz), electrographic status epilepticus (ESE), and high-frequency oscillations (HFOs) observed on intracranial EEG near the hematoma were reflected on scalp EEG in terms of appearance, timing, frequency, and amplitude.

**Results:**

Of the 116 patients, six developed intracranial hematoma and were included in the analysis. Focal RDA was observed on scalp EEG in all six cases, whereas intracranial RDA appeared earlier than focal RDA on scalp EEG. In all cases, spikes and polyspikes were superimposed on the RDA in the intracranial EEG, whereas these findings were observed on scalp EEG in only three cases. ESz, ESE, and HFOs were observed in all intracranial recordings but were not detected on scalp EEG.

**Significance:**

Focal RDA observed on scalp EEG during the acute phase may reflect growing epileptic activity and serve as a potential target for antiseizure medication therapy.

## Highlights

• Intracranial rhythmic delta activity (RDA) preceded scalp focal RDA in hematoma case.
• Intracranial epileptic activity was seen in all six cases, but in three by scalp EEG.
• Scalp focal RDA may indicate the need for antiseizure medication.

## 1 Introduction

Acute brain injuries such as traumatic brain injury, hypoxic encephalopathy, and stroke are often accompanied by epileptic activity, which can lead to secondary brain injury, hippocampal atrophy, cognitive decline, and eventually epileptogenesis (During et al., 1994; Herman, 2002; Vespa et al., 2010, Vespa et al., 2016; Gorter et al., 2015; De Marchis et al., 2016; Swissa et al., 2019; Aroniadou-Anderjaska et al., 2024; Cho and Hsieh, 2024). Electroencephalography (EEG) monitoring is essential for detecting epileptic activity during the acute phase of brain injury, particularly because non-convulsive status epilepticus (NCSE) cannot be recognized without an EEG examination, and prolonged NCSE may lead to long-term sequelae (Trinka et al., 2015; Sivaraju and Hirsch, 2023). Timely detection through EEG and prompt intervention with antiseizure medications (ASMs) are therefore strongly recommended (Brophy et al., 2012; Claassen et al., 2013; Abend et al., 2013; Dobesberger et al., 2015; Bitar et al., 2024).

The American Clinical Neurophysiology Society (ACNS) Standardized Critical Care EEG Terminology, most recently revised in 2021, provides standardized definitions for rhythmic and periodic patterns, incorporating considerations from the Salzburg Criteria for NCSE (Hirsch et al., 2013, Hirsch et al., 2021; Beniczky et al., 2013; Leitinger et al., 2015, Leitinger et al., 2016). Within this framework, rhythmic delta activity (RDA) is defined as the repetition of waveforms with a relatively uniform morphology and duration, without intervals between consecutive waveforms. The frequency range is specifically defined as 0.5–4.0 Hz. To be considered rhythmic, the duration of one cycle should vary by <50% compared with the subsequent cycle in over half of all consecutive cycles. Irregular or polymorphic delta waves were not considered as RDA. RDA is included in the ictal-interictal continuum only when it is lateralized (LRDA), averaging >1 Hz for at least 10 s and accompanied by a “plus modifier”. To what extent the LRDA recorded on scalp EEG reflects epileptic abnormalities occurring intracranially remains under investigation (Gaspard et al., 2013; Rodriguez Ruiz et al., 2017). In particular, to our knowledge, no reports have compared scalp and intracranial EEG recordings with focal RDA in the context of localized brain injury or intracerebral hemorrhage.

We encountered several patients with drug-resistant epilepsy who underwent intracranial electrode implantation for epileptogenic zone localization and subsequently developed postoperative intracranial hemorrhage. In these cases, RDA on intracranial EEG appeared adjacent to the hematoma and gradually evolved into electrographic seizures. Based on these observations, we retrospectively reviewed patients who developed intracranial hematoma following intracranial electrode implantation to assess how well focal RDA observed on scalp EEG reflects underlying epileptiform activity recorded intracranially. We also characterized the temporal evolution of epileptic activities using simultaneously recorded intracranial and scalp EEG.

## 2 Methods

### 2.1 Participants

We retrospectively reviewed cases from January 2003 to August 2024 in which simultaneous intracranial and scalp EEG recordings were performed for epileptogenic zone localization. We specifically analyzed patients who developed intracranial hematoma caused by electrode implantation. The surgical strategy, including the number and placement sites of stereotactic EEG (SEEG) electrodes, was determined by a multidisciplinary epilepsy surgery team comprising epileptologists (neurosurgeons, neurologists, pediatric neurologists, and psychiatrists), as well as rehabilitation and EEG specialists. This study was approved by the institutional ethics committee (approval number: 2020-761).

#### 2.1.1 EEG recordings

After inducing general anesthesia, all patients underwent craniotomy and/or burr hole surgery for the implantation of subdural grids, strips, depth electrodes and/or SEEG electrodes. All electrodes were manufactured by UNIQUE MEDICAL (Tokyo, Japan). Intracranial EEG monitoring was initiated immediately after surgery. Perioperative dexamethasone was not administered to prevent cerebral edema. The SEEG and depth electrodes used were either 1.2 (contact width, 2 mm; inter-contact spacing, 10 mm for electrodes with 5/6/8 contacts) or 1.5 (contact width, 2 mm; inter-contact spacing, 5 mm for electrodes with 12 contacts) mm in diameter. Contact numbers were numbered from the deepest point (designated as number 1). SEEG and depth electrodes were implanted employing stereotactic guidance systems such as VarioGuide® (Brainlab AG, Munich, Germany) or Autoguide (Medtronic, Dublin, Ireland). For subdural electrocorticography (ECoG), electrodes measured 5 mm in diameter with 10 mm center-to-center spacing (UNIQUE MEDICAL, Tokyo, Japan). Scalp EEG monitoring was performed simultaneously using disc electrodes placed according to the International 10–20 system. When surgical wounds interfered with electrode placement, electrodes were positioned as close as possible without disturbing the wound (Sakata et al., 2022). Scalp and intracranial recordings were referenced to a system electrode located within 10% of Cz and positioned away from the surgical wound. The typical sampling rate is 2 kHz; however, for some chronic subdural ECoG recordings, a 1-kHz sampling rate was used.

#### 2.1.2 EEG and clinical evaluation

EEG data were reviewed by two board-certified electroencephalographers and epileptologists (HS and TM), along with one board-certified electroencephalographer (AS). Only findings unanimously agreed upon by all reviewers were included in the analysis. Definitions of RDA, RDA + S (superimposed sharp waves or spikes, or sharply contoured), ESz (electrographic seizure), and ESE (electrographic status epilepticus) for intracranial recordings followed the 2021 version of the ACNS standardized critical care EEG terminology, originally developed for scalp EEG (Hirsch et al., 2021). In addition, the emergence of sustained RDA, appearing either continuously or intermittently in more than 80% of at least 1 min of recording, was defined as maximum RDA (max RDA).

#### 2.1.3 Frequency estimation method

RDA was identified in intracranial and scalp EEG recordings based on the ACNS criteria, requiring at least six consecutive rhythmic cycles. Over five samples were collected from each RDA episode, and frequencies were calculated using the NK Review Program (QP-112 A, Nihon Kohden). The highest observed frequency was recorded as the maximum frequency. In intracranial recordings, RDA frequency and amplitude varied over time. Once stable rhythmic activity was confirmed, the frequency was calculated based on the “max RDA” definition, as described above. The frequency of the scalp RDA was measured at the time corresponding to the appearance of the max RDA on intracranial EEG. High-frequency oscillations (130–150 Hz) were visually confirmed using an EEG trendgram (QP-160 A, Nihon Kohden). The frequency of the posterior dominant rhythm (PDR) of scalp EEG was measured during max RDA periods of intracranial recordings.

#### 2.1.4 Amplitude measurement

For both intracranial and scalp recordings, amplitudes were measured relative to the reference electrode of the corresponding recording system. In intracranial EEG, the maximum amplitude of the RDA was measured at its initial appearance and during max RDA. For intracranial spikes and polyspikes, five representative waveforms were selected for each case by visual inspection, and their amplitudes were measured for further analysis. Regarding scalp EEG, the maximum amplitudes of the scalp focal RDA and PDR during the max RDA periods of intracranial recordings were measured.

#### 2.1.5 Time course analysis

All time points were calculated relative to the end of the surgery. The appearance times of RDA, max RDA, ESz, ESE, spikes, and polyspikes were recorded.

### 2.2 Statistical analysis

To compare the appearance times of different EEG phenomena, the bootstrap method was used to assess whether the emergence order of intracranial RDA significantly differed from other findings (Python 3.13.7). Paired *t*-tests were used to compare amplitudes between intracranial and scalp recordings. The amplitudes of intracranial spikes and polyspikes were compared between cases with and without scalp-visible spikes or polyspikes using an exact permutation test.

## 3 Results

### 3.1 Clinical characteristics and EEG findings of the included patients

**Fig. 1:**
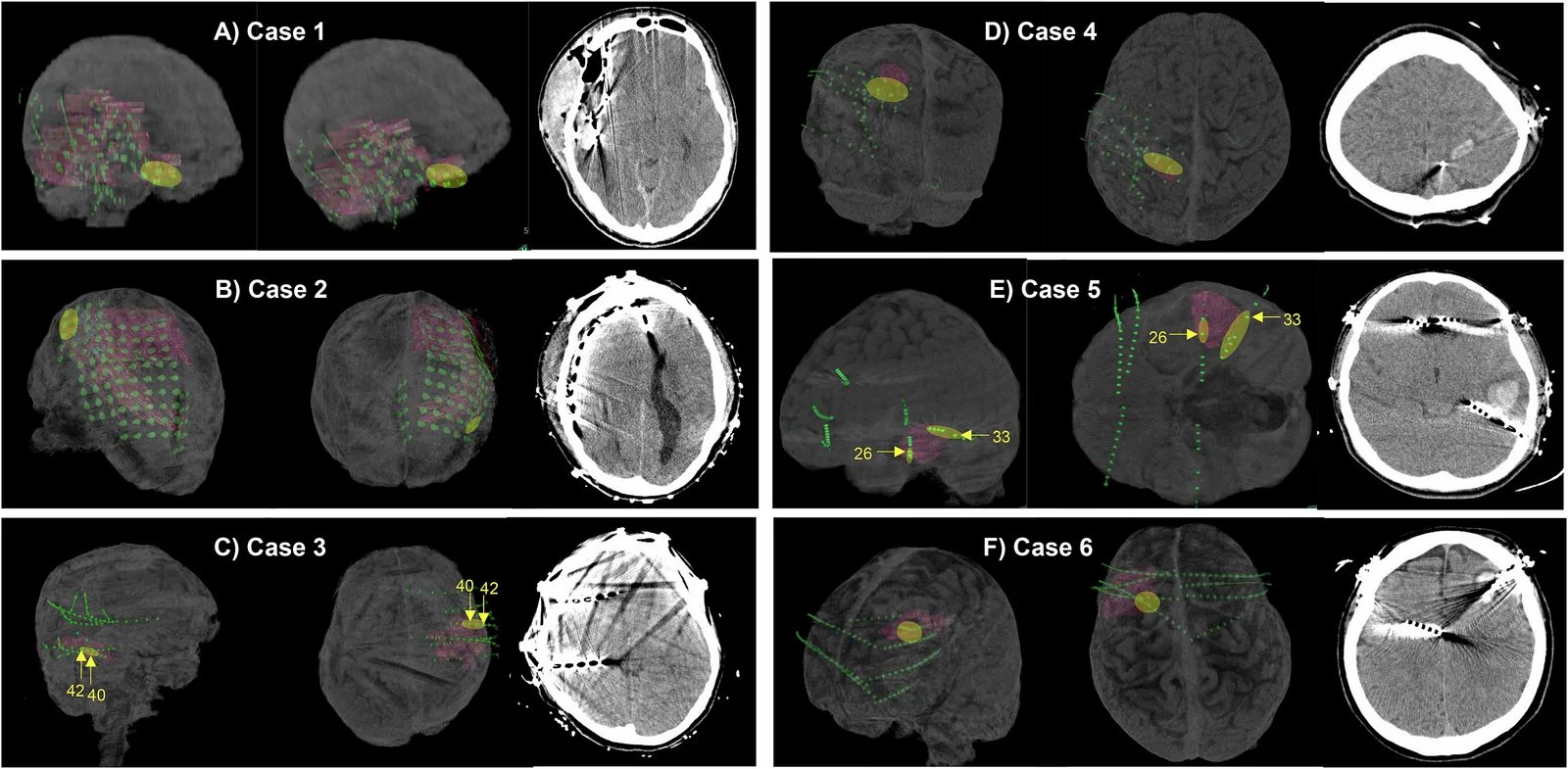
Locations of the electrode and hemorrhage period. Green indicates the electrodes, and red indicates the hematomas. The yellow areas indicate the regions where rhythmic delta activity was measured in the intracranial electroencephalography (EEG). The arrows and numbers in C) and E) refer to the stereo-EEG electrodes shown in Fig. 3, Fig. 4. (For interpretation of the references to colour in this figure legend, the reader is referred to the web version of this article.)

**Fig. 2:**
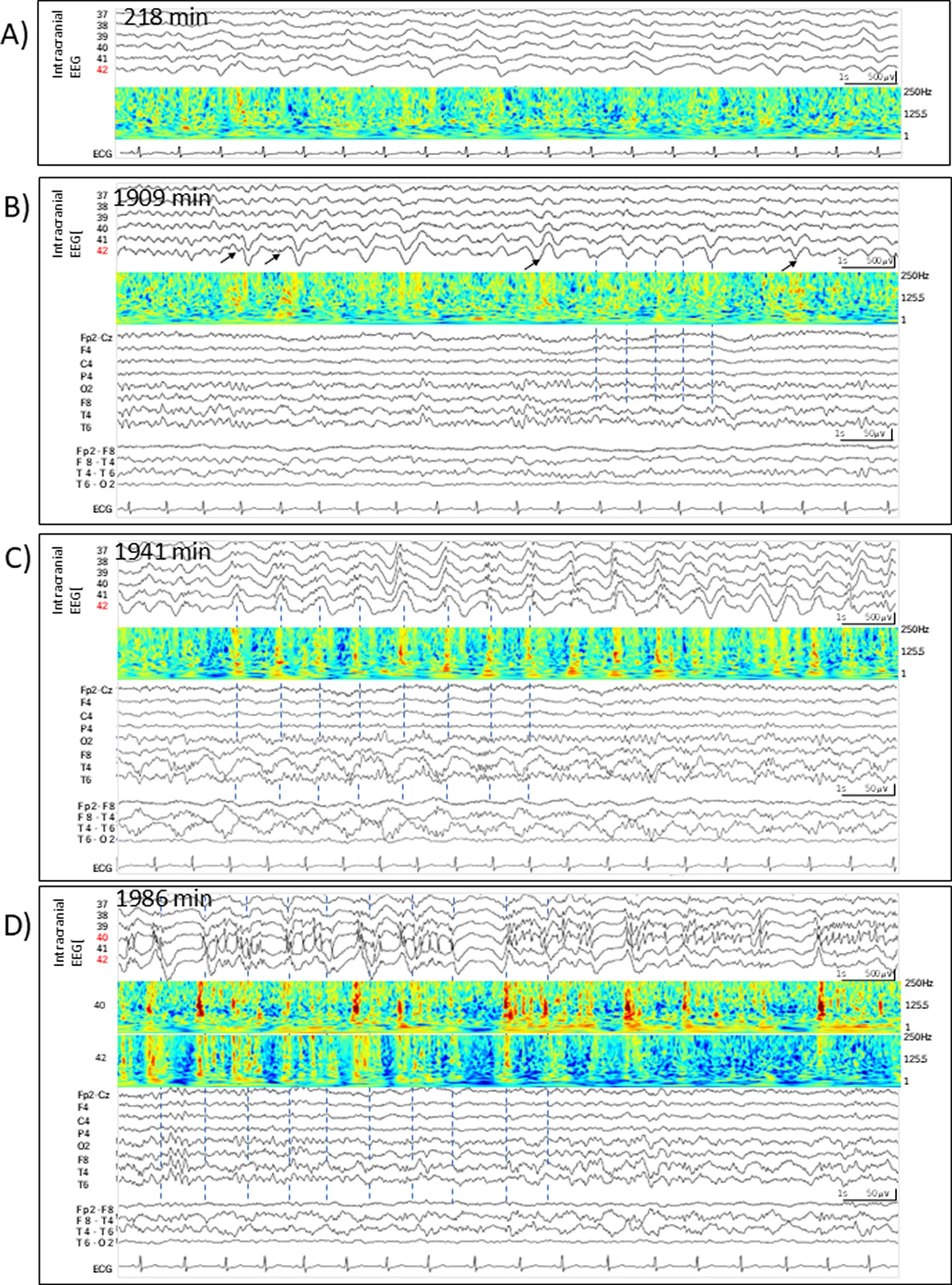
Time course of simultaneous intracranial and scalp electroencephalography (EEG) recordings in Case 3 period. Each panel shows, from top to bottom, intracranial EEG, the EEG trendgram, reference-montage scalp EEG near the hematoma, and the corresponding bipolar montage. The intracranial electrode numbers are arranged such that smaller numbers indicate deeper electrode positions. The EEG trendgram of the intracranial electrode highlighted in red is shown, and the location of the electrodes is illustrated in Fig. 1. Min indicates minutes elapsed since the end of surgery. A) At 218 min post-surgery, intermittent irregular delta waves without rhythmicity appeared on cortical electrodes (near the hematoma). Neither fast activity nor high-frequency oscillations (HFOs) were observed on EEG trendgrams. At this point, scalp electrodes has not yet been attached. B) At 1909 min post-surgery, irregular delta activity on intracranial EEG evolved into rhythmic delta at approximately 1.9 Hz, with superimposed fast activity (arrows). EEG trendgrams revealed HFOs at approximately 100–170 Hz. On scalp EEG, focal delta activity maximal at T4 was observed but lasted <10 s and lacked fast activity (dashed lines). C) At 1941 min post-surgery, spikes started to appear superimposed on rhythmic delta in intracranial EEG (RDA + S), with synchronized components in deeper electrodes. Some spikes were accompanied by fast activity. EEG trendgrams revealed HFOs in the 80–230 Hz range, along with gamma-band activity. On scalp EEG, focal rhythmic delta activity (RDA) maximal at T4 was observed, but spikes were absent (dashed lines). D) At 1986 min post-surgery, spike components over 4 Hz became superimposed on RDA in intracranial EEG, and HFOs at approximately 60–250 Hz were observed. On scalp EEG, relatively low-frequency rhythmic RDA was detected at T4 without spikes (dashed lines). (For interpretation of the references to colour in this figure legend, the reader is referred to the web version of this article.)

**Fig. 3:**
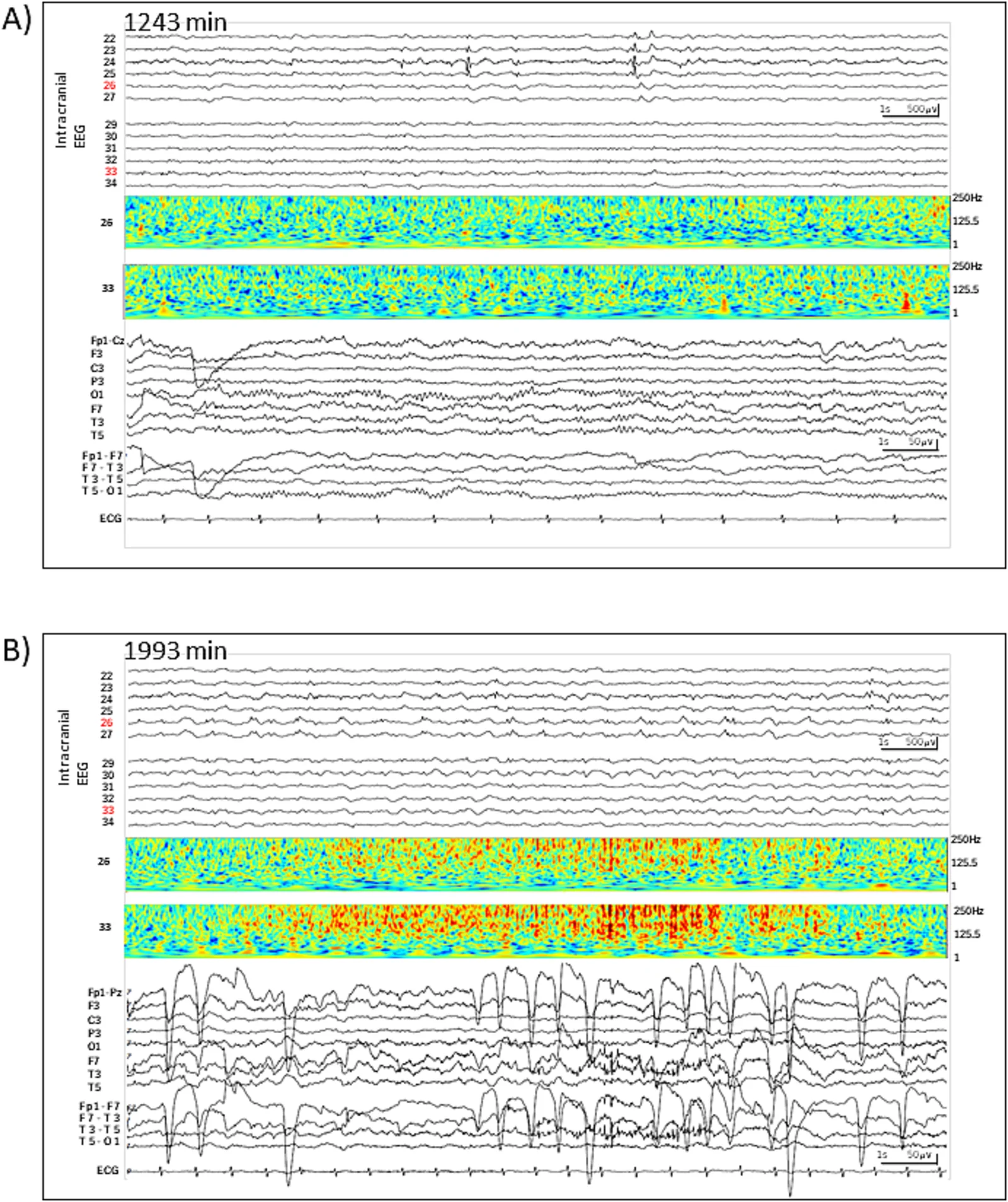

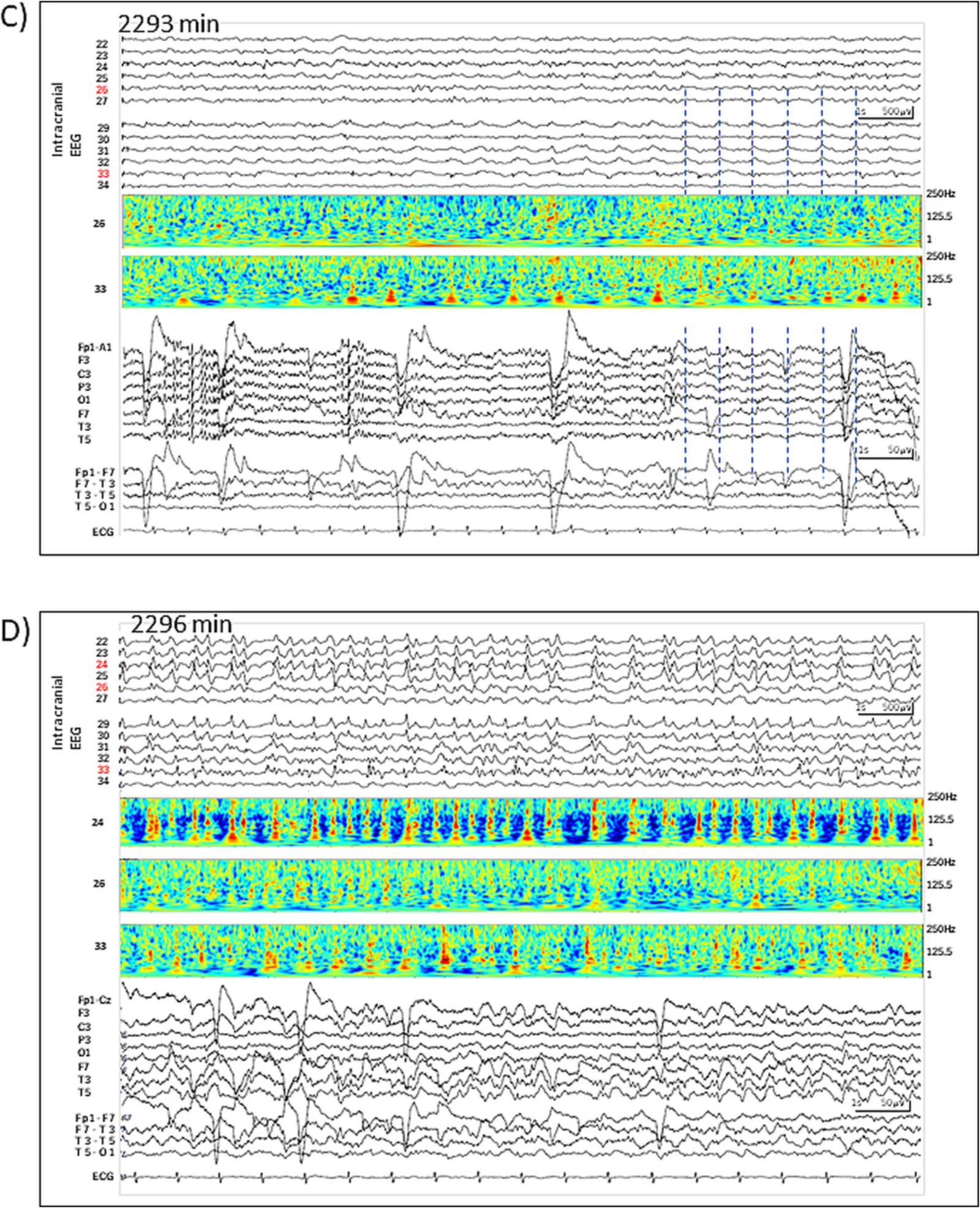
Time course of simultaneous intracranial and scalp electroencephalography (EEG) recordings in Case 5 period. Each panel shows, from top to bottom, intracranial EEG, the EEG trendgram, reference-montage scalp EEG near the hematoma, and the corresponding bipolar montage. The intracranial electrode numbers are arranged such that smaller numbers indicate deeper electrode positions. The EEG trendgram for the intracranial electrode highlighted in red is shown, and the location of the electrodes is illustrated in Fig. 1. Min indicates minutes elapsed since the end of surgery. A) At 1243 min after surgery, intracranial EEG revealed epileptic activity from the medial temporal lobe electrodes. No epileptic activity was observed in the other intracranial electrodes, no high-frequency activity was detected on EEG trendgrams, and no epileptic discharges were observed on the scalp recordings either. B) At 1993 min post-surgery, 1.3-Hz rhythmic delta activity (RDA) with superimposed spike components appeared in the intracranial EEG near the hematoma. EEG trendgrams revealed spike-synchronized activity in the 30–120-Hz band, separate from artifacts. Scalp EEG showed visible delta activity, but it lacked clear rhythmicity and fast components. C) At 2293 min post-surgery, widespread 1.7-Hz RDA with superimposed spikes (RDA + S) appeared in the intracranial EEG. EEG trendgrams showed spike-synchronized activity in the 30–120-Hz range. Scalp EEG showed focal delta activity maximally expressed at F7, but it was short in duration, and no spikes were observed (dashed line). D) At 2296 min post-surgery, repetitive spikes were recorded in intracranial EEG, corresponding to high-frequency oscillations on the trendgrams. Scalp EEG showed low-frequency rhythmic delta activity at F7 but again lacked spike components. (For interpretation of the references to colour in this figure legend, the reader is referred to the web version of this article.)

A total of 116 patients underwent simultaneous scalp and intracranial EEG recordings. Of these, 56 underwent chronic subdural ECoG only, 29 underwent SEEG only, and 31 underwent ECoG with depth electrode implantation. Among them, six patients developed intracranial hematoma and were included in the analysis. The six cases are listed in chronological order (Table 1). Three patients underwent reoperation (Cases 1, 2, and 4). One patient underwent hybrid implantation (subdural grid/strips plus stereotactic depth electrodes; Case 1), one underwent ECoG only (Case 2), and four underwent SEEG only (Cases 3–6) (Table 1). Two patients who developed subdural hematomas after ECoG implantation required urgent hematoma evacuation (Fig. 1A, B). In the four SEEG-only cases with intracerebral hematomas, ASMs were returned to their pre-monitoring dosages with close observation (Fig. 1C–F). The clinical courses of simultaneous intracranial and scalp EEG in Cases 3 and 5 are shown in Fig. 2, Fig. 3, respectively. In addition to the six patients included in this study, one other patient developed an intracranial hematoma associated with electrode implantation. However, this patient underwent emergency surgical evacuation of the intracranial hematoma before scalp EEG recording, precluding simultaneous scalp and intracranial EEG recordings; therefore, the patient was excluded from the present analysis. Intracranial EEG in this patient demonstrated RDA, RDA + S, ESz, and ESE. In contrast, none of the patients without intracranial hematoma exhibited RDA, RDA + S, ESz, or ESE.

**Table 1:** Patient demographic characteristics.

Case	Age	F/M	Onset (y.o.)	Estimated epileptogenic region	Intracranial recording	ASMs Before the operation	ASMs During monitoring	Hemorrhagic region	Treatment for hemorrhage	Epilepsy surgery	Seizure outcome (Engel's classification)
Case 1	20	M	6	Rt T, F	ECoG x 3 plates Depth x 2 electrodes	LEV 3000 CBZ 200 CLB 30	LEV 0 CBZ 0 CLB 30	Rt large subdural	Removal of the hematoma	Rt anterior temporal resection	II
Case 2	18	M	8	Rt T, F	ECoG x 4 plates	LTG 150 LCM 150	LTG 0 LCM 0	Rt large subdural	Removal of the hematoma	Rt SMA resection	V
Case 3	24	F	5	Rt T, F	SEEG x 8 electrodes	LEV 1000 LCM 100 ZNS 300	LEV 500 LCM 50 ZNS 150	Rt temporal	Observation Restart ASMs	Rt anterior temporal lobectomy	II
Case 4	11	M	7	Lt T, P	SEEG x 5 electrodes	LEV 2000 LCM 400	LEV 0 LCM 400	Lt parietal	Observation Restart ASMs	No resection	No resection
Case 5	14	M	10	Lt T, F	SEEG x 6 electrodes	VPA 1200 PER 400	LEV 0 LCM 0	Lt temporal	Observation Restart ASMs	No resection	No resection
Case 6	21	F	13	Lt F, Rt F	SEEG x 8 electrodes	LTG 200 LCM 200 PER 8	LTG 200 LCM 0 PER 0	Rt frontal	Observation Restart ASMs	Lt frontal lobectomy	Ia

Case numbers are arranged in chronological order. T, temporal lobe; F, frontal lobe; P, parietal lobe; aT, anterior temporal lobe; SMA, supplementary motor area; ECoG, electrocorticogram; SEEG, stereotactic intracranial EEG; LEV, levetiracetam; CBZ, carbamazepine; CLB, clobazam; LTG, lamotrigine; LCM, lacosamide; ZNS, zonisamide; VPA, valproic acid; PER, perampanel; ASMs, antiseizure medications.

### 3.2 Temporal profile of EEG findings

The appearance times of each EEG pattern on electrodes placed near the hematoma (intracranial RDA, max RDA, ESz, ESE, spikes, polyspikes, scalp focal RDA, and scalp focal RDA + S) are shown in Table 2. Intracranial EEG monitoring began 152.5 ± 501.3 min (median ± standard deviation [SD], range: 120–1124) postoperatively. In all cases, the intracranial EEG showed an RDA that fulfilled the ACNS criteria. In two cases (Cases 1 and 6), RDA was already present at the start of the EEG recording. For the remaining four cases, RDA appeared at 1672.5 ± 339.7 min (median ± SD, range: 1309–1993) postoperatively. Time to max RDA was 2076.5 ± 420.7 min (median ± SD, range: 1478–2635). All intracranial EEG recordings revealed ESz, ESE, and spikes. In two cases (Cases 1 and 6), these were already present at the start of monitoring. Excluding these two, ESz, ESE, and spike onset times were 1794.5 ± 219.9 min (median ± SD, range: 1627–2098), 1943.0 ± 166.0 min (median ± SD, range: 1877–2247), and 1773.0 ± 294.4 min (median ± SD, range: 1436–2098), respectively. Polyspikes were observed in all cases after the initiation of monitoring, with an onset time of 1993.0 ± 665.8 min (median ± SD, range: 1136–3036). High-frequency activity in the 30–250 Hz range was detected in all cases and superimposed on the spikes (Fig. 2, Fig. 3).

**Table 2:** Postoperative appearance time (minutes), frequency (Hz), and maximum amplitude (μV) of waves.

	Intracranial							Scalp		
Case	Start	RDA	maxRDA	ESz	ESE	Spike	Polyspike	Start	RDA	RDA + S
1	129	#	1478	#	#	#	1442	1083	#	1285
2	120	1436	2022	1673	1941	1436	1925	1213	1679	1947
3	176	1909	1941	1916	1945	1916	1941	1221	1937	2009
4	126	1309	2527	1627	1877	1630	3036	1273	2029	
5	1124	1993	2131	2098	2247	2098	2291	1124	2130	
6	1091	#	2635	#	#	#	1136	1091	#	
Median	152.5	1672.5	2076.5	1794.5	1943.0	1773.0	1933.0	1168.5	1983.0	1947.0
SD	501.3	339.7	420.7	219.9	166.0	294.4	665.8	78.7	193.3	401.3


	Intracranial					Scalp	Scalp	
	RDA		max RDA		Spike, Polyspike	RDA		PDR
Case	Frequency	Amplitude	Frequency	Amplitude	Amplitude	Frequency	Amplitude	Frequency
1	2.0	632.9	2.3	695.3	637.6	2.3	76.7	6.8
2	1.5	1700.0	1.8	1400.0	1560.0	1.8	114.0	7.4
3	1.9	532.2	1.2	1300.0	1068.4	1.2	71.3	8.1
4	2.3	1500.0	2.8	1200.0	1197.9	2.8	82.0	7.1
5	1.3	432.2	1.7	912.4	856.1	1.7	88.0	9.8
6	2.0	686.9	2.5	922.2	475.1	2.5	101.1	7.4
Median	1.9	659.9	2.1	1061.1	928.7	2.1	85.0	7.4
SD	0.4	542.2	0.6	270.5	376.0	0.6	16.0	1.1

Start: start time of the recording. RDA, rhythmic delta activity; ESz, electrographical seizure; ESE, electrographical status epilepticus; RDA + S, rhythmic delta activity plus spikes (sharp waves); PDR, posterior dominant rhythm # indicates activities that had already appeared by the time records began.

Scalp EEG recordings began 1168.5 ± 436.0 min postoperatively (median ± SD, range: 129–1273). Focal RDA was observed in all cases. In two cases (Cases 1 and 6), focal RDA was already present at the initiation of scalp EEG recording. In the remaining four cases, the onset time of focal RDA was 1983.0 ± 193.3 min (median ± SD, range: 1679–2130). The focal RDA was localized over the area of the hemorrhage on the scalp (Fig. 1, Fig. 2, Fig. 3). Focal RDA with superimposed spikes (focal RDA + S) were observed in three of six cases (Cases 1–3), with an onset time of 1947.0 ± 401.3 min (median ± SD, range: 1285–2009). No ESz or ESE was observed on the scalp recordings.

**Fig. 4:**
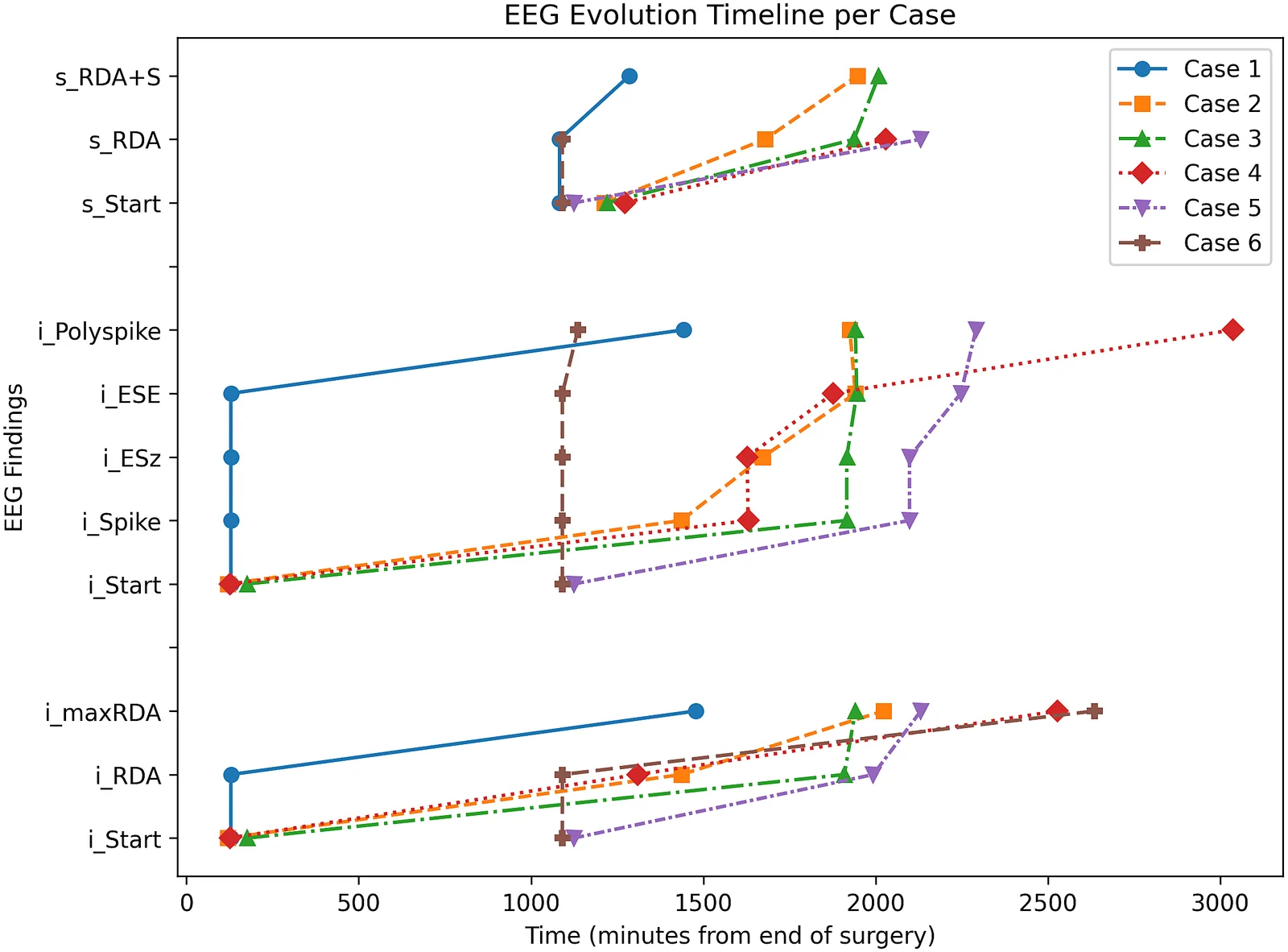
Appearance time from the end of surgery period. RDA, rhythmic delta activity; ESz, electrographic seizure; ESE, electrographic status epilepticus; RDA + S, rhythmic delta activity plus spikes (sharp waves).

Intracranial RDA appeared significantly earlier than other intracranial EEG findings, including max RDA, ESz, ESE, spikes, and polyspikes. It also occurred significantly earlier than focal RDA and focal RDA + S observed on scalp EEG (Table 2, Fig. 4).

### 3.3 Frequency and amplitude of intracranial and scalp focal RDA

Table 2 shows the frequency and amplitude of intracranial RDA, intracranial max RDA, and the amplitude of scalp focal RDA. The frequency of scalp RDA was identical to that of the intracranial max RDA. Intracranial RDA frequency at its initial appearance (Cases 2–5) or first detection (Cases 1 and 6) was 1.9 ± 0.4 Hz (median ± SD, range: 1.3–2.3). Although the frequency decreased in one case (Case 3), it increased in the remaining five. The max RDA frequency was 2.1 ± 0.6 Hz (median ± SD, range: 1.2–2.8). No significant difference was observed between the frequencies of the initial/first-detected intracranial RDA and intracranial max RDA (*p* = 0.299).

Intracranial RDA amplitude at initial appearance or first detection was 659.9 ± 542.2 μV (median ± SD, range: 432.2–1700.0), while that of intracranial max RDA was 1061.1 ± 270.5 μV (median ± SD, range: 695.3–1400.0). No significant difference in amplitude was observed between the initial/first-detected intracranial RDA and the intracranial max RDA (*p* = 0.407).

The amplitude of scalp focal RDA at the time of intracranial max RDA was 85.0 ± 16.0 μV (median ± SD, range: 71.3–114.0), which was significantly lower than the intracranial amplitude of 1061.1 ± 270.5 μV (median ± SD, range 695.3–1400.0) (*p* < 0.001), approximately one-twelfth in magnitude.

### 3.4 Comparison between cases with and without scalp-visible spikes and polyspikes

The median intracranial spike/polyspike amplitude was 928.7 ± 376.0 μV (median ± SD, range: 449.6–1700.0) (Table 2). In Cases 1–3, in which spikes/polyspikes were visible on scalp EEG, the median amplitude was 1100.0 ± 398.1 μV (median ± SD, range: 530.0–1700.0), whereas in Cases 4–6, in which spikes/polyspikes were not visible on scalp EEG, it was 844.2 ± 319.5 μV (median ± SD, range: 449.6–1400.0). Although the median amplitude was higher in the scalp-visible group, the difference was not statistically significant (*p* = 0.5).

### 3.5 PDR

The PDR was preserved even during periods of sustained intracranial max RDA, with a frequency of 7.4 ± 1.1 Hz (median ± SD, range: 6.8–9.8) (Fig. 2, Fig. 3, Table 2).

### 3.6 Clinical seizures attributable to ESz and ESE

Mild consciousness impairment and incomplete hemiparesis were observed in two cases of subdural hemorrhage following chronic subdural ECoG placement (Cases 1 and 2). However, no patient exhibited any apparent clinical seizure symptoms attributable to ESz and ESE.

### 3.7 Clinical course

In Cases 1 and 2, who underwent ECoG, emergency hematoma evacuation was performed together with resection of the regions showing habitual seizure activity. In the remaining four cases, intravenous fosphenytoin was administered, and the doses of anti-seizure medications were restored to their original levels, resulting in the resolution of RDA, RDA + S, ESz, and ESE. In two of these cases (Cases 3 and 6), the regions responsible for the habitual seizures were subsequently resected. In the other two cases (Cases 4 and 5), resective surgery was not performed because the epileptogenic zone responsible for the habitual seizures could not be sufficiently localized (Table 1).

## 4 Discussion

In this case series, simultaneous intracranial and scalp EEG recordings in six patients with intracranial hematoma demonstrated that focal RDA on scalp EEG consistently overlaid clearly identifiable epileptic activity on intracranial EEG, including spikes, polyspikes, ESz, ESE, and HFOs. Intracranial RDA appeared earlier than focal RDA on scalp EEG, and most epileptic features visible on intracranial EEG were either absent or markedly attenuated on scalp EEG. These observations provide direct empirical support for the view that focal RDA on scalp EEG during the acute phase of brain injury can serve as a surrogate marker of underlying intracranial epileptic activity.

Scalp EEG recordings are affected by volume conductors, such as the scalp, subcutaneous tissue, skull, and cerebrospinal fluid, which attenuate signal amplitude to approximately one-tenth of its original strength by the time it reaches the scalp surface (Hashiguchi et al., 2007). Moreover, fast activity and spikes detectable on intracranial EEG are often attenuated and may not be visible on scalp EEG (Hashiguchi et al., 2007; Sakata et al., 2022; Mukae et al., 2023). Consistent with previous studies of patients with acute brain injury, intracranial EEG showed a higher signal-to-noise ratio than scalp EEG and allowed earlier spike detection (Waziri et al., 2009; Mikell et al., 2016; Witsch et al., 2017; Fernández-Torre et al., 2021; Yuan et al., 2024). Our cases showed that ESz, ESE, and HFOs detected on intracranial EEG were not observed on scalp EEG, and spike components were detected on the scalp in only three of the six cases. No difference in intracranial spike amplitude was found between patients with and without scalp-visible spikes or polyspikes. Although the spatial extent of epileptic activity cannot be accurately determined using SEEG and therefore this interpretation remains speculative, our previous study suggested that the detectability of spikes and polyspikes on scalp EEG depends on spike amplitude and on the spatial extent of the underlying epileptic activity (Hashiguchi et al., 2007). Clearly detectable RDA on intracranial recordings in our cases may not have been recorded as focal RDA on scalp EEG because of reduced amplitude or shorter duration. Therefore, it is important to recognize that spike activity present on intracranial EEG may be attenuated on scalp EEG, leaving only the background rhythmic slow-wave activity visible, thereby leading to the appearance of focal RDA resembling temporal intermittent RDA (Di Gennaro et al., 2003; Fox et al., 2022).

In this study, the frequency of RDA first observed on intracranial recordings was 1.9 ± 0.4 Hz, and in five of the six cases, the frequency increased over time, reaching 2.1 ± 0.6 Hz. Lateralized PDs (LPDs) were associated with seizure risk across all frequencies, whereas LRDA and generalized PDs were associated with increased seizure risk when the frequency exceeded approximately 1.5 Hz (Rodriguez Ruiz et al., 2017). All six of our cases progressed to ESz or ESE on intracranial recordings, and the RDA was sustained over time. Witsch et al. (2017) reported that during PDs at frequencies >2 Hz, cerebral blood flow (CBF) increased to meet the elevated metabolic demand, and that in the absence of further increases in CBF, tissue hypoxia can occur, potentially leading to the failure of cerebral compensatory mechanisms. Epileptic activity around hematomas associated with SEEG implantation has also been reported (Diab et al., 2022; Mahizhnan et al., 2022), and intracortical injection of penicillin in animal models induces spike-and-wave discharges in the cortex (Fisher and Prince, 1977a; 1997b). Taken together with our findings, these reports suggest a possible vicious cycle wherein focal RDA arises from local cortical tissue injury, leading to increased energy demand to sustain RDA activity, which in turn exacerbates tissue injury and promotes further persistence of focal RDA.

The epileptogenicity of high-frequency activity remains controversial. However, some studies have suggested that activity >70 Hz detected on intracranial EEG during acute brain injury may be of epileptogenic significance (Mikell et al., 2016). In our intracranial recordings, high-frequency activity started to emerge concurrently with RDA appearance. These high-frequency oscillations became phase-locked to the rhythmic periodicity of the RDA, eventually evolving into spike activity. Although high-frequency activity might already be present because of underlying epileptogenicity in our patient cohort, its emergence in synchrony with RDA suggests that fast activity and spike discharges may arise through mechanisms similar to those that generate focal RDA.

Previous reports involving simultaneous scalp and intracranial EEG recordings have primarily focused on hemispheric patterns, such as LPD or LRDA. To our knowledge, no prior studies have specifically investigated focal RDA in the manner presented in this report. The closest related concept to focal RDA is brief potentially ictal rhythmic discharges (BIRDs), defined as rhythmic activity >4 Hz lasting ≥0.5 to <10 s (Yoo et al., 2014). In a subsequent series of 3520 patients with epilepsy, BIRDs and paroxysmal fast activity were observed in 3% of patients, all of whom had recorded seizures, with the BIRDs location corresponding to the scalp seizure-onset zone (Yoo et al., 2021). Our findings extend these observations to the acute, post-injury setting and demonstrate that focal RDA, rather than fast rhythmic activity, can serve as a scalp signature of evolving intracranial epileptic activity.

Regarding the presence of electroclinical seizures, the clinical symptoms observed in two patients (mild consciousness impairment and incomplete hemiparesis in Cases 1 and 2) were most plausibly attributable to the hematoma itself or to compression-induced effects rather than to clinical seizures, and differed from their habitual seizures. Therefore, these events could not be classified as electroclinical seizures.

Recent work has shown that, among patients with acute brain injury, persistent or worsening LPD or LRDA is associated with poor prognosis (Narrett et al., 2024). Similarly. among older patients with status epilepticus, those with PDs tend to have poorer outcomes (Yoshimura et al., 2016), and the prognosis of status epilepticus depends more on background EEG activity than on rhythmic or periodic features (Alvarez et al., 2015). In the present cases, the scalp RDA was localized, the background rhythm was preserved, and patients were under medical supervision; thus, their prognosis was not poor. Although early intervention with ASMs is desirable when epileptic activity is detected, the pharmacological burden may itself contribute to mortality in patients with compromised general condition (Trinka et al., 2023). Therefore, when initiating treatment with antiseizure or sedative medications, performing a comprehensive assessment that includes EEG findings and the patient's overall systemic condition is crucial.

## 5 Limitations

Because all patients had epilepsy, their brains may have been more susceptible to epileptic activity, potentially limiting the generalizability of these findings to individuals with acute brain injury but without preexisting epilepsy.

## 6 Conclusion

In six patients who developed focal intracranial hematoma during electrode implantation for epileptogenic zone localization, focal RDA was observed on scalp EEG. Intracranial RDA appeared earlier than focal RDA on scalp EEG. Simultaneous intracranial EEG recordings revealed spikes and polyspikes in all six cases; however, these were detectable on scalp EEG in only three cases. Furthermore, ESz, ESE, and HFOs were recorded in all cases on intracranial EEG but were not detected on scalp EEG. These findings suggest that focal RDA may reflect underlying epileptic activity and that treatment with ASMs should be considered. Future studies should validate these findings in larger cohorts of patients with acute brain injury without preexisting epilepsy.

The following are the supplementary data related to this article.

**Supplementary Figure S1:**
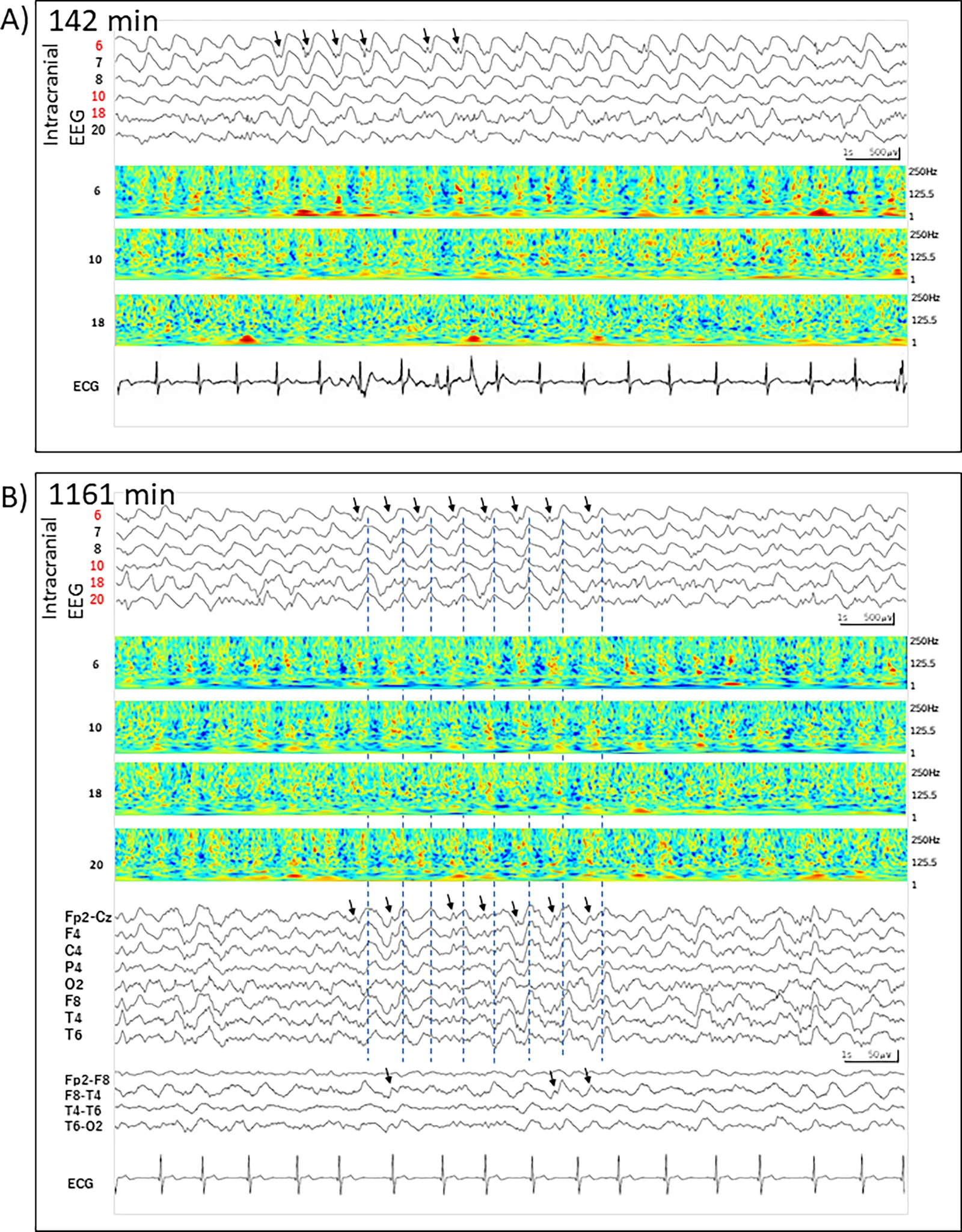

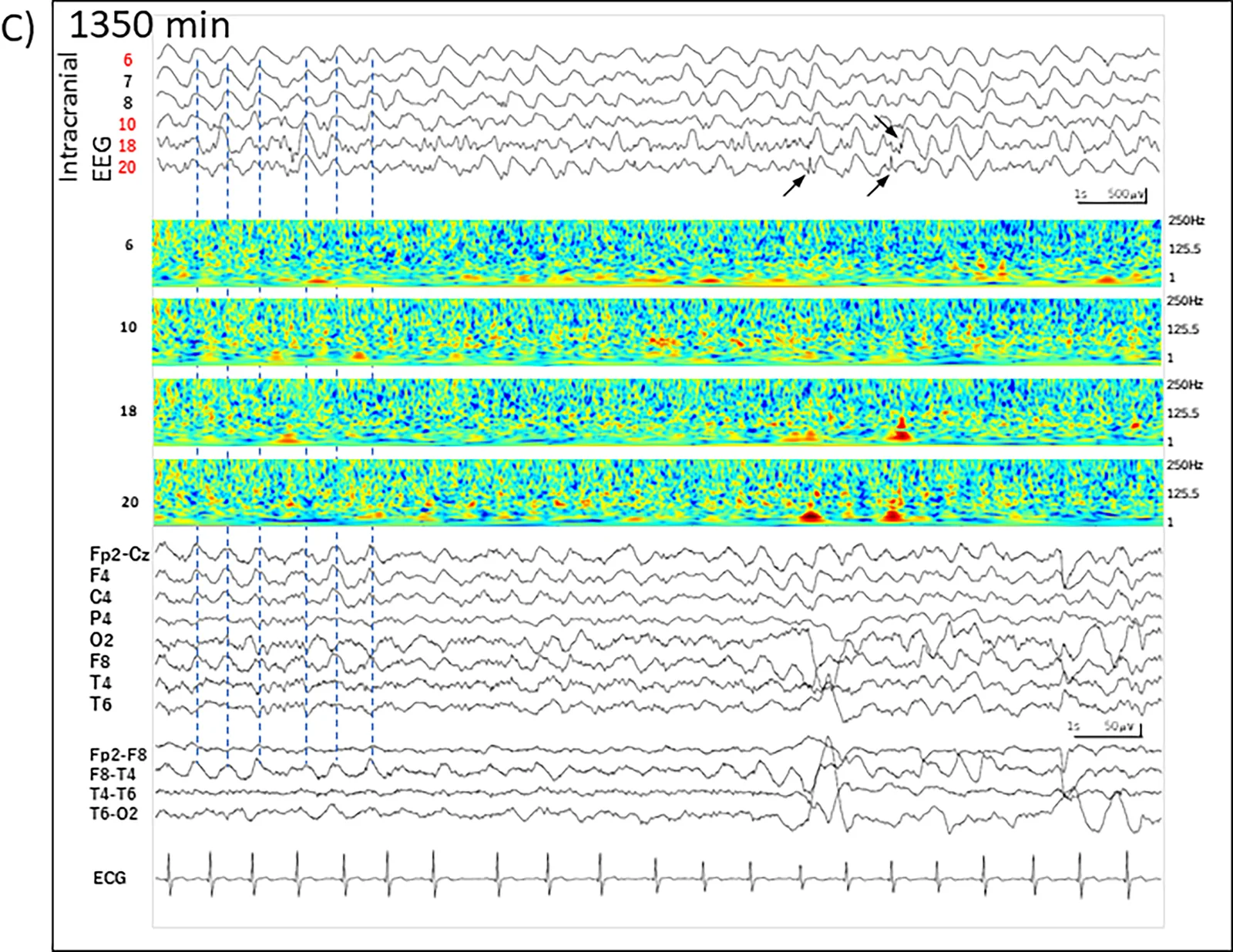
Time course of simultaneous intracranial and scalp electroencephalography (EEG) recordings in Case 1

Each panel shows, from top to bottom, intracranial EEG, the EEG trendgram, reference-montage scalp EEG near the hematoma, and the corresponding bipolar montage. The intracranial electrode numbers are arranged such that smaller numbers indicate deeper electrode positions. The EEG trendgram for the intracranial electrode highlighted in red is shown, and the electrode locations are illustrated in Fig. 1. Min indicates minutes elapsed since the end of surgery. A) At 142 min post-surgery, 2.0 Hz rhythmic delta activity was observed on the cortical electrodes near the hematoma. Spikes appeared in the form of spike and wave complexes (black arrows). Fast activity and high-frequency oscillations (HFOs) were observed on the EEG trendgrams and were time-locked to the spikes. At this point, scalp electrodes had not yet been attached. B) At 1161 min post-surgery, rhythmic delta activity was also observed on right anterior scalp EEG (dashed line). When the amplitude of spikes recorded from intracranial electrodes 6 and 7 exceeded approximately 700 μV, corresponding spikes became visible over the right anterior hemisphere on the scalp EEG (black arrows). However, because the duration was too short, the findings did not meet the criteria for RDA + S. C) At 1350 min postoperatively, a spike was observed on one of the intracranial electrodes, but no corresponding spike was detected on the scalp EEG.

**Supplementary Figure S2:**
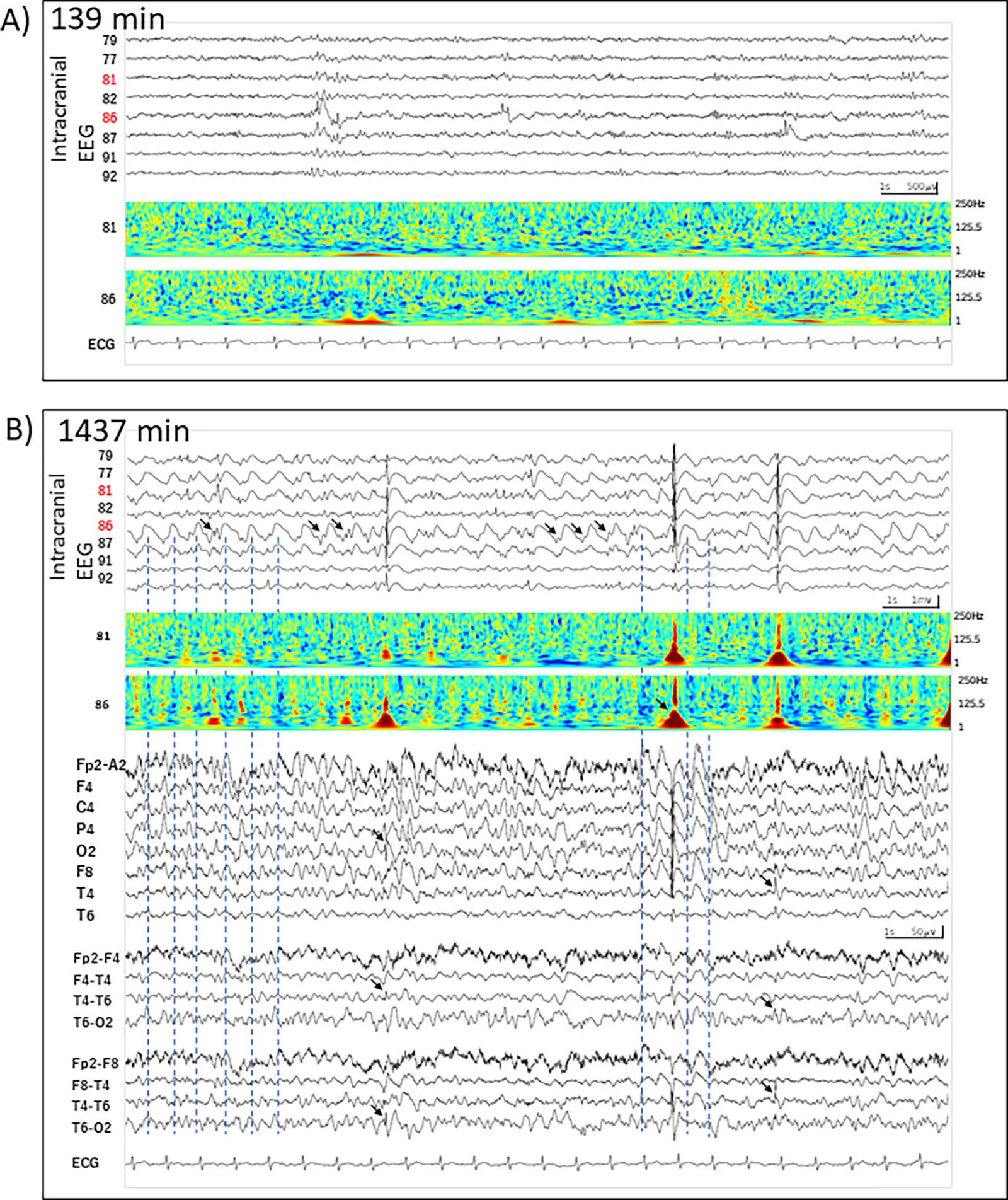

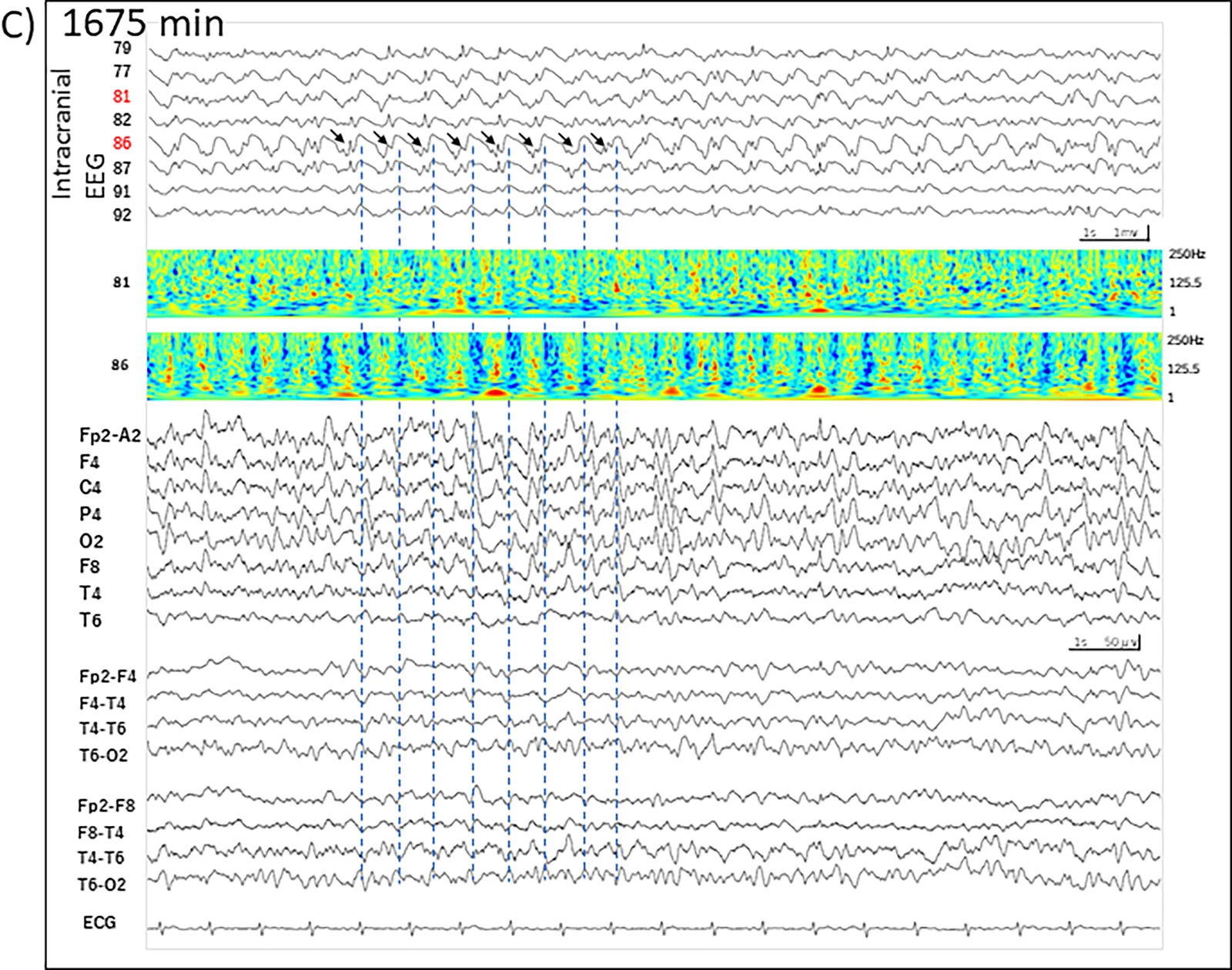
Time course of simultaneous intracranial and scalp electroencephalography (EEG) recordings in Case 2

Each panel shows, from top to bottom, intracranial EEG, the EEG trendgram, reference-montage scalp EEG near the hematoma, and the corresponding bipolar montage. The intracranial electrode numbers are arranged such that smaller numbers indicate deeper electrode positions. The EEG trendgram for the intracranial electrode highlighted in red is shown, and the electrode locations are illustrated in Fig. 1. Min indicates minutes elapsed since the end of surgery. A) Rhythmic delta activity was not observed on the intracranial EEG. Scalp electrodes were not attached at this point. B) Rhythmic delta activity at 1.5–1.8 Hz accompanied by spikes appeared on intracranial channel 86 (black arrows). Activity in the 80–130 Hz range was observed on the EEG trendgrams and was time-locked to the spikes. Although corresponding rhythmic delta activity was observed on scalp EEG, its duration was too short to satisfy the criteria for RDA. Intracranial spikes with amplitudes exceeding 2 mV and wide distribution were detectable on the scalp EEG. C) Continuous rhythmic delta activity, predominantly at 1.8 Hz and reaching its maximum amplitude, was recorded on intracranial channel 86. The spikes occasionally appeared as spike and wave complexes (black arrows). Activity in the 80–150 Hz range was observed on the EEG trendgrams and was time-locked to the spikes. When the intracranial rhythmic delta activity involved a broader cortical area, it could occasionally be detected on scalp EEG; however, its duration remained too short to meet the criteria for RDA. No spikes were detected on scalp EEG. D) Compared with panel C), the spikes of intracranial EEG became larger in amplitude and more prominent (black arrows). The scalp EEG showed 1.8-Hz RDA + S; however, the component corresponding to the intracranial spikes were less sharply contoured than those recorded on the intracranial EEG (black arrows).

**Supplementary Figure S3:**
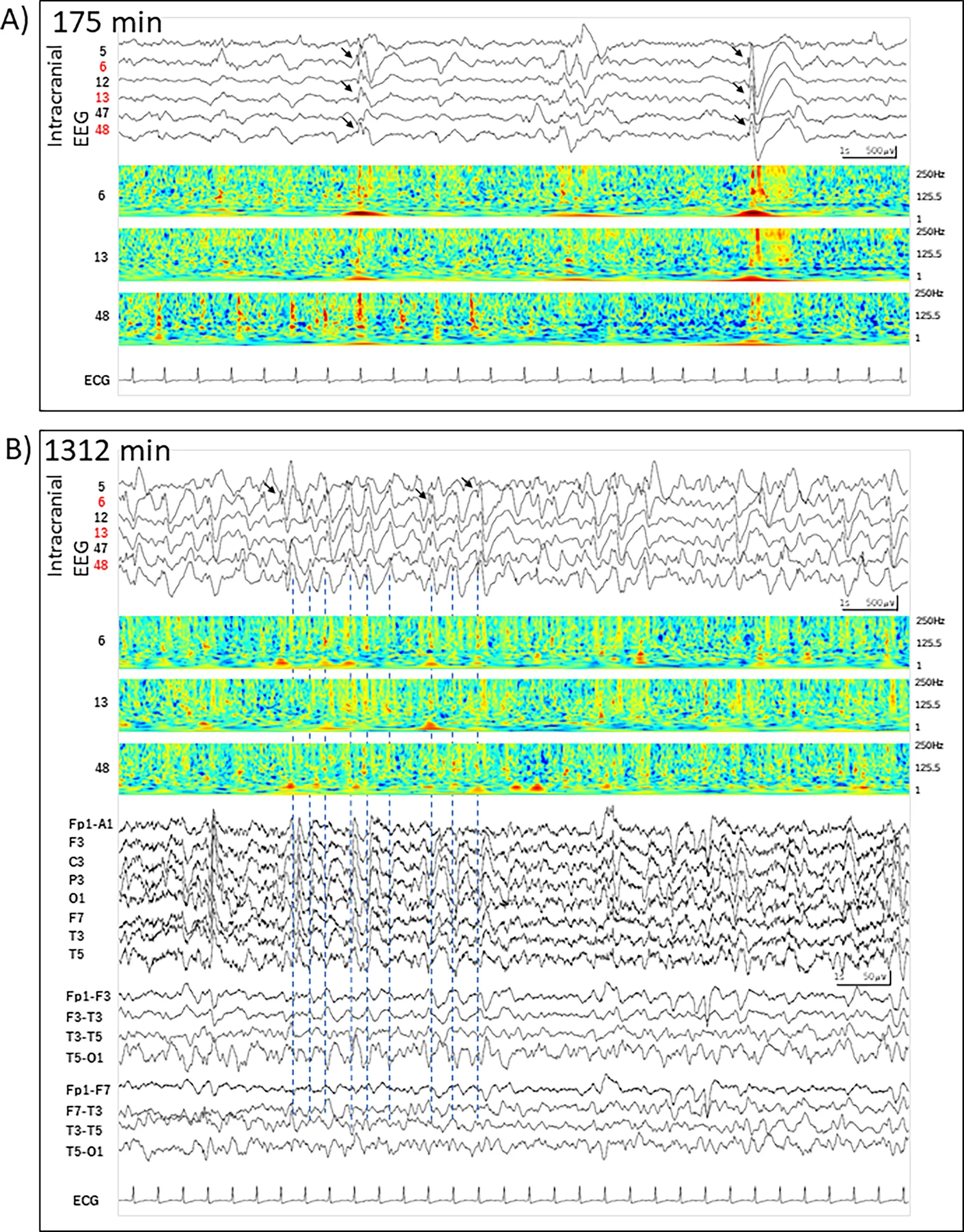

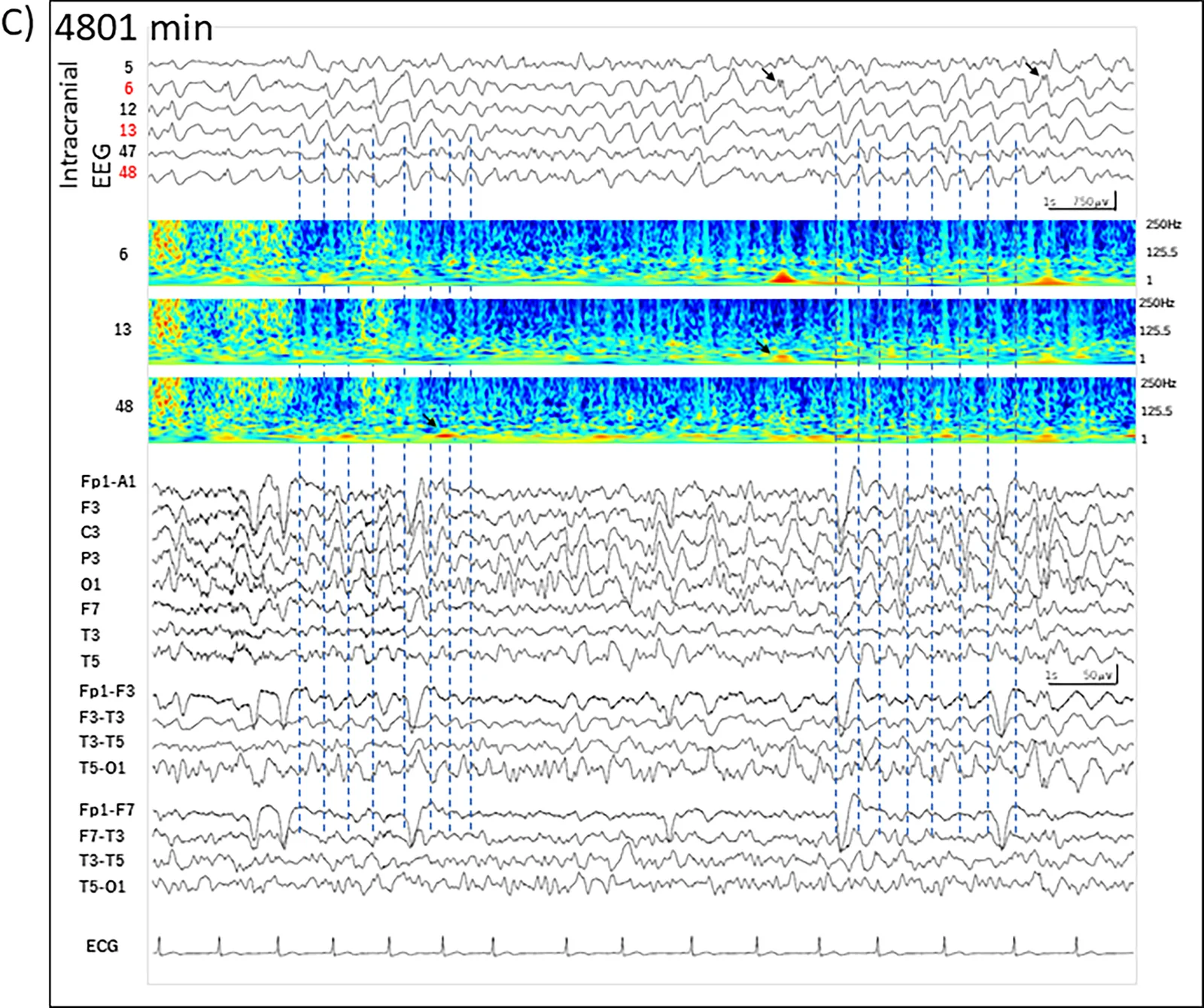
Time course of simultaneous intracranial and scalp electroencephalography (EEG) recordings in Case 4

Each panel shows, from top to bottom, intracranial EEG, the EEG trendgram, reference-montage scalp EEG near the hematoma, and the corresponding bipolar montage. The intracranial electrode numbers are arranged such that smaller numbers indicate deeper electrode positions. The EEG trendgram for the intracranial electrode highlighted in red is shown, and the electrode locations are illustrated in Fig. 1. Min indicates minutes elapsed since the end of surgery. A) Intermittent spikes were observed on intracranial channels 6, 13, and 48 (black arrows). Activity in the 80–110 Hz range was time-locked to the spikes on EEG trendgram. Although delta activity was present on intracranial channel 48 (convexity side), rhythmic delta activity was not observed. At this time point, scalp electrodes had not yet been attached. B) Rhythmic delta activities at 2.3 Hz appeared on the intracranial EEG, with superimposed spikes. Activity in the 50–250 Hz range was observed on the EEG trendgra, and was time-locked to the spikes (black arrows). Rhythmic delta activity was also observed on scalp electrode T5, corresponding to activity recorded from the intracranial electrode on the convexity side (channel 48); however, its duration was too short to satisfy the criteria for RDA. No spikes were observed on scalp EEG. C) Intracranial EEG showed continuous 2.8-Hz rhythmic delta activity. Fast activity was superimposed on channel 6, although it was not clearly visible on the EEG trendgram (black arrows). Scalp EEG showed intermittent rhythmic delta activity; however, its duration was too short to meet the criteria for RDA. Simultaneous ECG recording demonstrated irregular RR intervals.

**Supplementary Figure S4:**
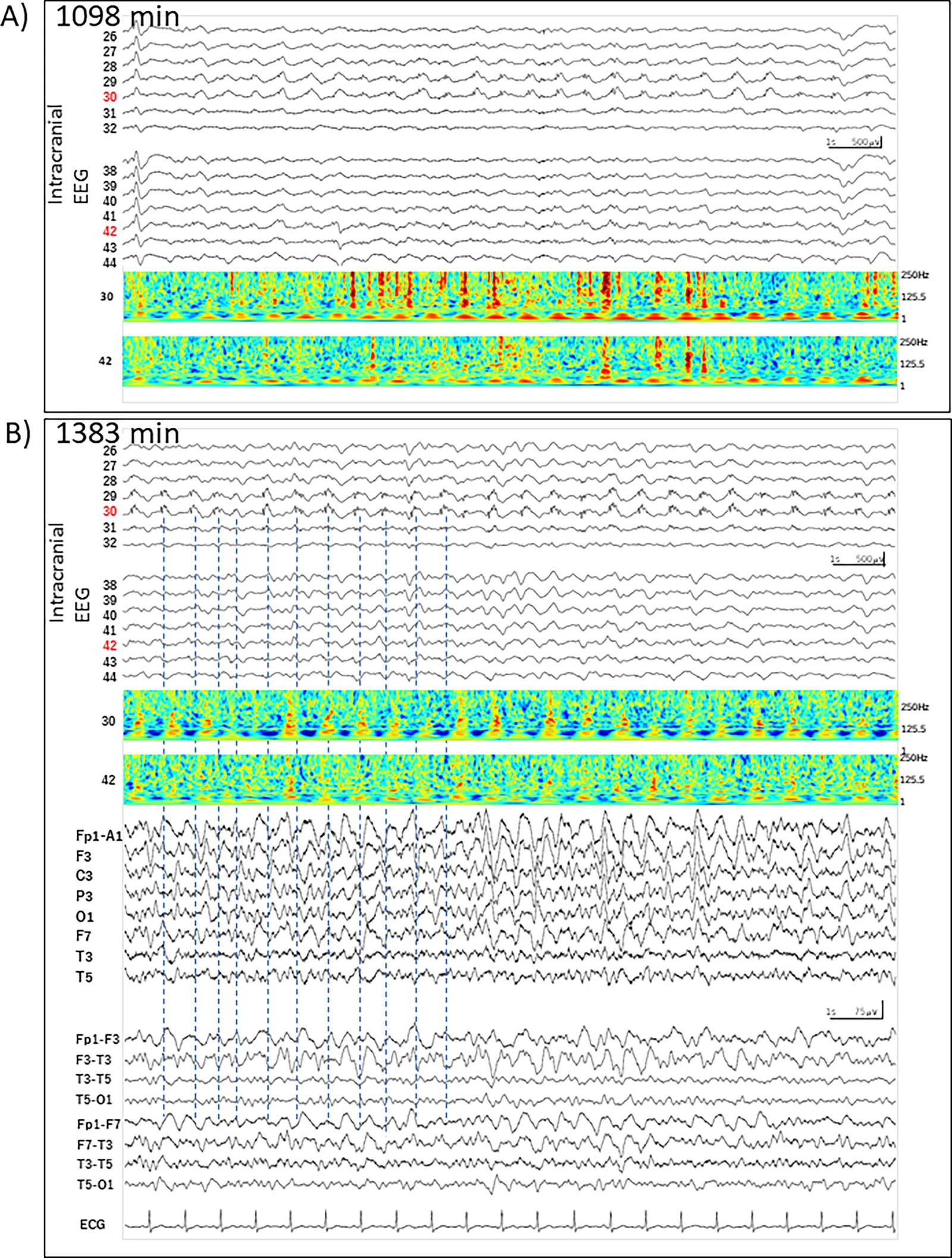
Time course of simultaneous intracranial and scalp electroencephalography (EEG) recordings in Case 6

## Author contributions

Ayumi Sakata: Conceptualization, data curation, formal analysis, investigation, methodology, visualization, writing the original draft, reviewing, and editing. Kimiaki Hashiguchi, Takato Morioka: Patient management; data collection. Noriko Isobe, Koji Yoshimoto, and Yuya Kunisaki: Data curation, supervision, writing, review, and editing. Kazuhisa Kuwabara and Kou Matsumoto: Patient management, data collection, and figure preparation. Nobutaka Mukae, Takafumi Shimogawa, Takahiko Mukaino: Patient management; data collection; formal analysis; writing, review, and editing. Hiroshi Shigeto: Conceptualization, data curation, formal analysis, investigation, methodology, visualization, writing the original draft, writing the review and editing, funding acquisition, and supervision. Eriko Watanabe: Data curation; formal analysis. Takahiro Yamaguchi, Taira Uehara, Toshiki Okadome, Yasunari Sakai, Yuri Sonoda, Satoshi Akamine, Chong Pin Fee, Kenta Kajiwara: Patient management and data collection.

## Funding

This work was partially supported by the 10.13039/501100001691Japan Society for the Promotion of Science (JSPS) KAKENHI Grant-in-Aid for Scientific Research (C) (Grant Number 23K06930).

## Ethical publication statement

We confirm that we have read the journal's guidelines on issues involved in ethical publication and affirm that this report is consistent with these guidelines.

## Declaration of generative ai and ai-assisted technologies in the manuscript preparation process

None.

## Declaration of competing interest

H.S. has received speaker honoraria from the UCB, Japan. None of the authors declare any conflicts of interest.
